# Navigating techno-stress: A qualitative exploration of university faculty's experiences and perspectives in the Peruvian context amidst the return to classes and the post-COVID-19 era

**DOI:** 10.12688/f1000research.141432.2

**Published:** 2024-10-01

**Authors:** Josefina Amanda Suyo-Vega, Monica Elisa Meneses-La-Riva, Víctor Hugo Fernández-Bedoya

**Affiliations:** 1 Grupo de Investigación “Educación Virtual, Universidad Cesar Vallejo, Los Olivos, Lima, 15314, Peru

**Keywords:** Technology, Teaching and learning, Qualitative study, Techno-stress, Adapting to technology, Work overload, Mental well-being

## Abstract

Background: Technology serves as a potent tool that enhances the quality of teaching and learning experiences. However, when educators lack proficiency in utilizing technology, it leads to obstacles in providing effective education, resulting in emotions of frustration, diminished self-assurance, and uncertainty regarding their teaching abilities. This study aims to investigate the experiences of university faculty members in relation to the impact of technology on their mental well-being.

Methods: For this qualitative study, ten professionals engaged in university-level teaching, encompassing research domains, were selected for interviews. Inclusion criteria were based on teaching experience, tenure, specific courses or subjects taught, and active involvement during the research phase. The guiding question for the study was framed as follows: "What are the experiences and perceptions of university faculty members concerning techno-stress?" Additionally, the study identified four subcategories: work overload, social and emotional interaction, adaption to new technologies, and expectations and teaching quality.

Results: Techno-stress is a composite of emotional and physical reactions triggered by the improper use of technology. Faculty members’ encounters with techno-stress have substantial implications for their quality of life. The subcategories shed light on different aspects of the faculty’s experiences, including the burden of excessive workload, the influence of social and emotional interactions, the process of adapting to evolving technologies, and the connection between expectations and the quality of their teaching.

Conclusions: This research underscores the significance of technology in higher education, highlighting its potential to positively impact teaching and learning. Nevertheless, faculty members’ struggles with techno-stress indicate a pressing need for effective training and support. Establishing limits on connectivity with technology and others emerges as a crucial step in maintaining a healthy work-life balance. Ultimately, addressing techno-stress and providing appropriate guidance are essential for safeguarding the well-being of university faculty members and, consequently, enhancing the overall educational experience.

## Introduction

The digital era brings changes in how teachers teach, and students learn. While technology is a useful tool, it also has negative impacts on the mental health of teachers.

University professors now create and manage more digital content, leading to increased stress and anxiety ( Alvites, 2019; Rivadeneira Guerrero *et al.,* 2020). Additionally, in the university setting, professors must continuously update their knowledge on technology-related topics, which generates pressure ( Alvites, 2019). Students expect quick responses and constant availability from their professors to address even minor doubts ( Suyo-Vega *et al.,* 2022). It is necessary for teachers to establish boundaries on working hours and workload to protect their mental health and well-being. Factors like social isolation, digital fatigue, heavier workloads, and adaptation to new technologies negatively impact the mental health of professors ( Abarca *et al.*, 2022; De Lima Santana *et al.*, 2022; Morales-Rodríguez, 2021). Therefore, practical strategies must be implemented to improve their well-being ( CEPAL, 2020).

Studies on the incorporation of ICTs in class sessions show that older professors, aged over 45, tend to prefer traditional methods, finding them more satisfying and committing to the teaching process ( Genimon Vadakkemulanjanal *et al*., 2021). In India, research on technostress confirms a negative relationship with technology among different age groups and levels of commitment based on experience and age ( Genimon Vadakkemulanjanal *et al.*, 2021). Specifically, technostress is generated by the use of technology in academic activities carried out by teachers ( Zheng *et al*., 2022). Similarly, the teaching staff indicates that when experiencing various levels of technostress, they employ different strategies to mitigate it ( Khlaif *et al.*, 2023c).

Considering the impact of the pandemic on the job stress of university professors, researchers emphasize the importance of addressing this issue in higher education to ensure the well-being of teachers. They suggest measures to prevent and reduce the negative impact of stress ( Manzur-Vera *et al.,* 2022).

The technostress or tech stress is caused by work overload and the process of adapting to technologies ( Tondeur *et al.,* 2008), affecting the interpersonal relationships of professors with students, colleagues, friends, and family ( Maslach *et al.*, 2001; Romero-Martín & Fraile-Aranda, 2017; Torres-Hernández, 2023).

The literature review on technostress reveals four subcategories that are the focus of this research, based on Maslach’s Burnout theory and Tondeur’s work on the relationship between work and technology.

The first subcategory, Work Overload, describes the excessive workload and the constant need to be available, answering emails, WhatsApp messages, and engaging in social media interactions. Professors respond to this overload in two ways: some seek help to complete their tasks, while others reject assistance and prefer to handle all activities, despite spending excessive hours in front of a computer. Studies suggest paying attention to technostress and its effects on teachers’ quality of life and work-life balance ( Decataldo & Fiore, 2022). Prolonged use of electronic devices and exposure to blue light can affect the physical health of professors, leading to eye fatigue, headaches, and sleep disturbances ( Estrada Araoz *et al.,* 2021; Fernández Villacres *et al*., 2021; Kaya, 2020). It is essential to take care of professors’ physical and cognitive health, as it can impact their attention, concentration, and memory ( Corrente *et al.*, 2022; Jimenez, 2021). Therefore, professors must be mindful of using technology effectively and in a balanced manner.

While some studies demonstrate the positive impact of Information and Communication Technologies (ICT) on the work experience, they also suggest that appropriate implementation of ICT can reduce demands. Thus, considering individual characteristics and organizational procedures in implementing ICT can improve how teachers interpret their experiences related to ICT and ultimately enhance their performance and well-being at work ( Pansini *et al.*, 2023; Sánchez-Macías *et al.*, 2021).

The second subcategory, Social and Emotional Interaction, deals with how teachers manage and channel emotions in front of students. Focusing on emotions in a digital environment is challenging, as they are complex human characteristics that are difficult to define, recognize, and classify ( Francisti *et al.*, 2023). Research emphasizes the importance of social and emotional interaction to avoid negative emotional and behavioral consequences ( Simona *et al.*, 2021). Therefore, teachers seek diverse pedagogical methods and practices to strengthen students’ competencies ( Zotova *et al.*, 2021). Identifying emotions, sharing feelings, and developing empathy towards others are vital components of the learning process, or at least they aid in it ( Ch’ng, 2019; Drewelow, 2020; Wetcho & Na-Songkhla, 2022).

The third subcategory, Adaptation to New Technologies, describes the experiences of both teachers and students, as the post-pandemic generation faces technological changes from multiple perspectives. Advantages and opportunities arise from the use of technological tools in the teaching process ( Bendito Cañizares & Sánchez Botas, 2021; Pribeanu & Gabriel Gorghiu, 2022; Shulga *et al.*, 2021). However, research conducted in Turkey reveals that both teachers and students prefer in-person teaching, as they detected issues with distance learning ( Rakhimgalieva *et al.*, 2021).

Finally, the fourth subcategory, Expectations and Teaching Quality, deals with different teaching approaches, some focused-on theory, others on practice, and some aiming to combine both. However, technology plays a role in all cases. Despite two years passing since the pandemic, there is a lack of attention and concrete actions to address teacher technostress, affecting the social, occupational, and psychological aspects ( Abarca *et al.*, 2022). The intention is to adapt to technology without harming the mental health of teachers or students, in order to provide quality education, recognizing the potential of ICTs ( Sánchez-Macías *et al.*, 2021). The relevance of using tools like Moodle and Facebook is evident as they improve student learning and teaching quality ( Delgado-García *et al.*, 2017).

With regard to gender, there are various research studies related to university students as well as female professors, which show high levels of stress in relation to the male gender ( Garcés-Delgado *et al.*, 2023; Mena Freire *et al.*, 2022). These findings highlight the need for in-depth research on technostress in the academic context in order to expand our understanding of the experiences and challenges faced by professionals in teaching.

Moreover, the presence of technology in the workplace can lead to anxiety, frustration, and emotional distress, especially when faced with various platforms or technological tools in daily tasks. Additionally, technostress can manifest in different ways, such as feeling overwhelmed by excessive information, constant interruptions from emails, lack of control over technology, and difficulty in maintaining balance and setting boundaries between work and personal life ( Wang *et al.*, 2016; Wang *et al.*, 2008).

Finally, it is necessary to regulate emotions to mitigate technostress, thus enhancing a more positive perception of technological tools. Training and professional development sessions for teaching staff should include emotional regulation strategies to manage stress ( Castellanos-Alvarenga *et al.*, 2024). Furthermore, open communication between professionals and policy makers should be fostered to improve the academic work environment ( Khlaif, Khalili, Affouneh, *et al.*, 2023a; Khlaif, Sanmugam, & Ayyoub, 2023b).

The following research question was proposed: How does technostress manifest among university professors, particularly in terms of work overload, challenges in social and emotional interactions, adaptation to new technologies, and changing expectations?

The objectives set were to analyze the experiences and perceptions related to (a) Work overload, (b) Social and emotional interaction, (c) Adaptation to new technologies, and (d) Expectations and quality of teaching.

As there is a need to delve deeper into the topic of technology and its impact on mental health, it is essential to investigate the experiences and perceptions of university professors regarding technostress. This research is relevant as technology becomes more prevalent in education and may be affecting the mental health of teachers, which, in turn, can influence students’ academic success. This investigation aims to offer new knowledge and strengthen existing theories to find solutions to the identified problems.

## Methods

### Ethical considerations

The research obtained approval from the ethics committee of the doctoral program at Universidad César Vallejo (N° 00035-2023), visible at: https://doi.org/10.5281/zenodo.8226629.

The research adopted a qualitative approach and employed the grounded theory method, which enables the researcher to conduct organized and rigorous research ( De la Espriella & Gómez Restrepo, 2020; Vives Varela & Hamui Sutton, 2021). This investigation was conducted within the constructivist paradigm, allowing key participants or interviewees to construct their experiences related to a phenomenon ( Guba & Lincoln, 1994). In this regard, the research focuses on interpreting how teaching professionals make sense of their experiences with technology.

Eight (8) female interviewees and two (2) male interviewees were selected. The choice to interview ten key participants was based on the fact that, for an in-depth interview, a minimum of ten participants is recommended ( Hernández-Sampieri & Mendoza Torres, 2018). This decision is grounded in the gender inequality within higher education and research in Peru ( Nieto Montesinos *et al.*, 2019). Despite the increased participation of women as full-time professors, their representation in high-level academic roles remains low ( UNESCO - IESALC, 2021). The proportion of female researchers is also significantly lower compared to the total number of female professors ( Chávez Irigoyen & Penelas Ronso Merino, 2021). The gender distribution in the interviews aims to gain a better understanding of this issue and give voice to the experiences of both women and men in higher education and research.

The key participants in the research were university teachers with prior and post-pandemic teaching experience. The selection was made among professors from both national and private universities who had more than five years of experience. This restriction was due to the fact that these professionals had engaged in both in-person and virtual teaching activities before and after the pandemic. The chosen university professors taught courses related to research, including seminars, methodology, thesis design, and development. In all cases, these professionals were responsible for reviewing students’ projects and theses, which required dedicating numerous hours to reading and revising each academic product.

For data collection, verbal informed consent was obtained from the professors, and dates and times for interviews were scheduled. Since each interviewed teacher is in different regions of Peru, consent was given during the interview sessions, which were recorded.

**Figure 1.**
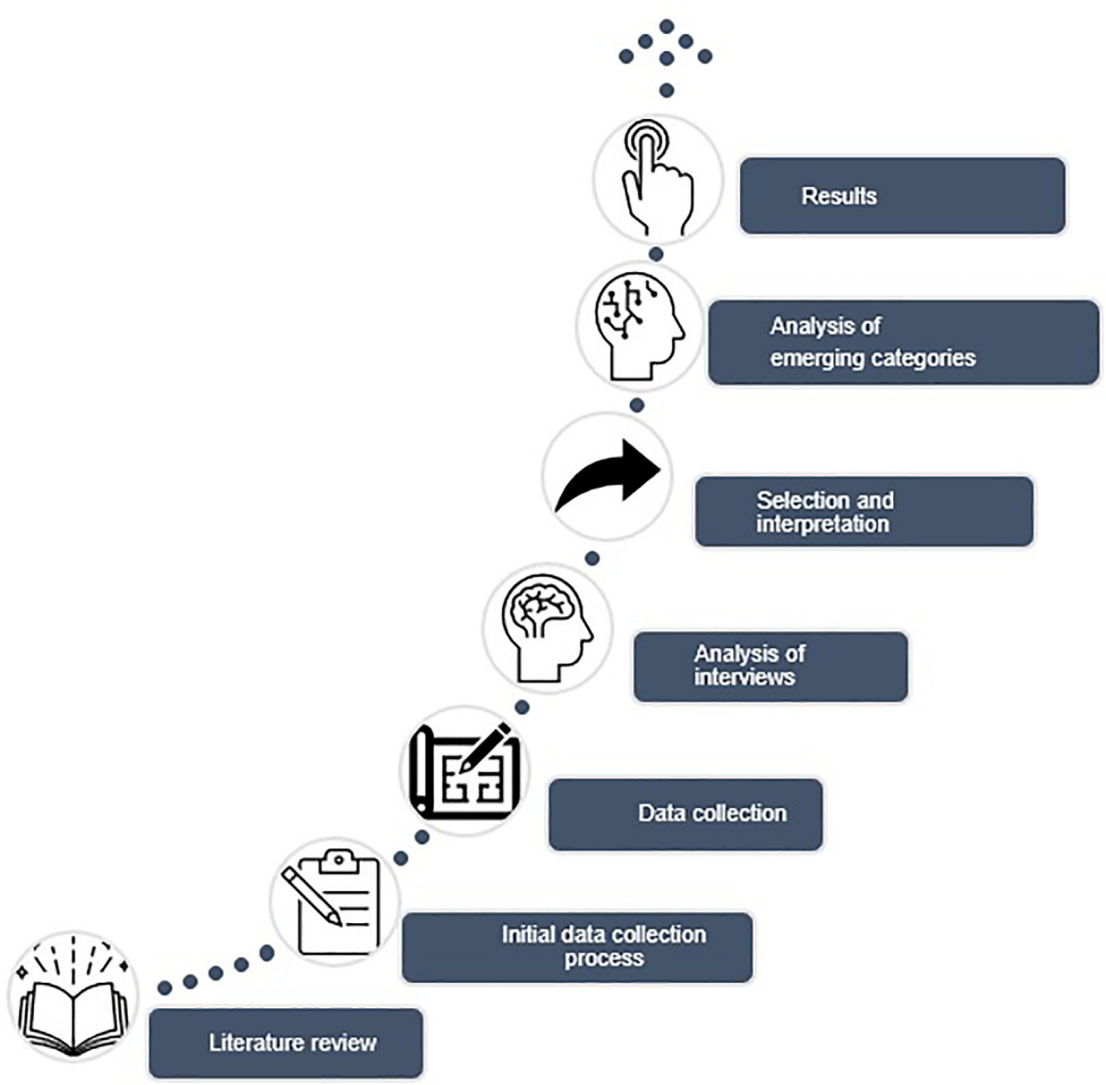
Methodological processes in the research.

The interview type was semi-structured, as the questions were provided in advance but were adjusted based on the interviewees' responses in order to enable an in-depth analysis ( Díaz-Bravo *et al.*, 2013). The interview period spanned four months, from April 2023 to August 1, 2023. Each interview lasted an average of 31 minutes, during which four important aspects were discussed: (a) Work overload, including technical difficulties and quality expectations. (b) Social and emotional interaction, encompassing physical and mental health issues. (c) Adaptation to new technologies, addressing the challenges of adjusting to emerging technologies. (d) Expectations and teaching quality. These four subcategories were derived from the literature review and are described in Table 1, detailing each statement’s characteristics. Following the interviews, a meeting was held with five researchers from the team to discuss and analyse the interviewees’ responses and establish new subcategories. No specialized software was used for data processing. Finally, the results and conclusions were written, as shown in Figure 1.

**Table 1.** Category, subcategories, statements, and questions about technostress.

Category	Subcategories	Statements	Questions
Technostress	Work overload	Description of the current workload compared to other moments in your work life.	1.- How would you describe your current workload compared to other periods of your professional life?
		Exploration of feelings when asking for help when feeling overwhelmed.	How do you feel about asking for help when you feel overwhelmed with your work?
		Possibility of delegating tasks to colleagues.	Do you think there is any type of work that you could delegate or share with your colleagues?
	Social and emotional interaction	Description of the relationship with the students.	How would you describe your relationship with your students?
		Management of emotions in the face of stress.	How do you manage your emotions when you feel stressed or frustrated?
		Strategies that teachers use to take care of their emotional health while carrying out their teaching duties.	What strategies do you use to take care of your mental and emotional health while working as a teacher?
	Adaptation to new technologies	Updating in technologies to improve teaching skills.	How do you stay updated on new technologies to enhance your skills and performance as a teacher?
		Description of the integration of technologies in the teaching process.	How do you integrate technologies into your teaching methodology?
		Analysis of beliefs about technology that changes the way of teaching.	How do you think technologies will change the way teaching and learning take place in the future?
	Expectations and quality of teaching	Description of teaching in the university.	How would you describe your teaching approach at the university?
		Personalization of teaching according to the needs of university students.	How do you personalize your teaching to meet individual needs, especially considering the diversity and heterogeneity of university students?
		Evaluation of the learning and progress of university students.	How do you assess your students' learning and progress at the university?

Table 1 presents the statements for the interviewees, derived from the subcategories resulting from the analysis of the literature review.

The second instrument used was the group interview, which derived from the technique of Group Discussion. In the virtual context, a meeting was conducted with five education professionals who discussed the stated positions. The questions were guided as shown in Table 1, as obtaining depth was best achieved by listening to each professional’s viewpoint based on their experience.

For the application of the instrument, it was analyzed and validated through education professionals. Additionally, it met the criteria of quality (a) Credibility, (b) Transferability, (c) Reliability and Confirmability, and (d) Reflexivity ( Korstjens & Moser, 2018).

In the research process, a meticulous data processing stage was conducted, enabling the management of data in a rigorous and reliable manner. Table 2 describes the strategies and activities carried out in accordance with the established quality criteria. The activities undertaken align with strategies such as interaction with the study subjects, data triangulation, member checking, detailed description, audit trail recording, and journaling.

**Table 2.** Activities carried out according to quality criteria.

Criteria	Strategies	Activities conducted
Credibility	Interaction with study subjects	The university faculty was personally and voluntarily invited to participate in the research, either through a phone call or, in some cases, via WhatsApp for virtual communication. The interview guide and informed consent were sent before the interview. Finally, the interview was conducted through a virtual meeting.
	Data triangulation	After obtaining the information from the faculty, a critical analysis was performed on each response, comparing it with the background information and theoretical references. The information collected was strengthened during the development of the discussion chapter.
	Member checking	Each participant was identified with a code that is recognizable to the research team. Additionally, the length of service and teaching experience before and after the pandemic were taken into account. These data served as references to establish generalizations in the new knowledge.
Transferability	Detailed description	The responses of the interviewees were transcribed by the research team, adhering to scientific rigor, including (a) autonomy, allowing each interviewee to provide their responses even if they were superficial, and follow-up questions were not possible for the same question; (b) beneficence, ensuring that the interviewees responded freely without any harm, and assuring them that all information shared was solely for research purposes; (c) justice, where all interviewees were subject to the same requirements to be part of the research. The responses of each interviewee were transcribed and analyzed. One practice involved searching for keywords, identifying the most frequently mentioned terms considered relevant by the team.
Reliability and Confirmability	Audit log	For the analysis, relevant articles were selected from impact-indexed databases such as Scopus, SciELO, Web of Science, and ERIC to provide support for the Discussion section. The participants’ responses were coded using the initials of their gender and age, for example, M55, M45, F56, F58, F41, F36, F46, F43, F52, F65. For instance, “M” represents male and “55” represents the age. Additionally, each participant was assigned a subject number such as S1, S2, S3, S4, S5, S6, S7, S8, S9, and S10. These codifications can be observed in Table 3. Furthermore, recordings of each interview were saved for reference.
Reflexivity	Diary	The findings were shared with the research team through Google Drive, with the purpose of conducting a critical analysis. Each link contains the recording of each interviewee.

**Table 3.** Audit log of the education professional interviewed.

Subject number	Gender	Age	Degree	Courses that he or she usually teaches	Years of university experience	Subject code
S1	Female	55	Bachelor of Education	Thesis Development, Research Methodology	10	S1F55
S2	Female	55	Bachelor of Nursing	Thesis Development, Thesis Project, Research Methodology	5	S2F55
S3	Male	53	Bachelor of Education	Biostatistics, Research Methodology	10	S3M53
S4	Female	58	Bachelor of Philosophy	Thesis Development, Thesis Project, Research Methodology	8	S4F58
S5	Female	49	Bachelor of Education	Thesis Development, Research Methodology	23	S5F49
S6	Female	58	Bachelor of Economics	Thesis Development, Thesis Project	15	S6F58
S7	Male	38	Systems Engineer	Biostatistics, Research Methodology	10	S7M38
S8	Female	59	Bachelor of Nursing	Research in Hospital Settings	17	S8F59
S9	Female	55	Bachelor of Education	Thesis Development	9	S9F55
S10	Female	55	Bachelor of Education	Research Methodology	7	S10F55

Table 3 describes the number of subjects, gender, the teacher's specialization, the course taught, years of service, and the coding assigned to key participants. The column labeled 'gender' was included to identify the potential influence of this factor in the results section, as well as to adhere to scientific rigor in transparency and the SAGER guidelines recommendation to disaggregate by sex ( Heidari *et al.*, 2016). Furthermore, the selection of teachers who teach research at the postgraduate level was based on criteria of experience and relevance in university teaching. Similarly, qualified higher education faculty members who were willing to contribute to the study were contacted.

For the data analysis stage, a specific methodological approach was followed, allowing for the identification of emerging themes based on responses provided by key participants. The coding of the interviewees and their responses strengthened the identification of subcategories. Tables 4, 5, 6, and 7 provide detailed coding of the interviewees, their responses, and the emerging concepts, which were used to draw conclusions and develop a profound understanding of the techno-stress-related topic. Furthermore, we applied the SAGER guidelines to discern the analysis between individuals of male and female gender ( Heidari *et al.*, 2016).

**Table 4.** Responses of the interviewees on workoverload.

Subject code	Gender	Work overload	Emerging concepts
S1F55	Female	“…There’s more mental than physical strain. Before the pandemic, it was physical fatigue. Today, I feel the work overload is more balanced…”	Mental and physical workload in balance.
S2F55	Female	“…Moderate work overload, I take on my responsibilities. In the past, I could delegate without worries, but today my academic responsibilities do not allow me to delegate my tasks…”	Moderate workload and responsibilities without delegation.
S3M53	Male	“…Before the pandemic, there was normal stress, but during the pandemic, we had to learn to manage technology, and it was stressful…”; “…teachers have to learn to manage their time effectively…”; “…due to health reasons…”	Stress due to new knowledge in technology and information communication systems (TICS).
S4F58	Female	“I easily adapted to the new technological tools and improved material organization.”	Easy adaptation.
S5F49	Female	“…The timings got confused…”; “…much more workload, meetings outside working hours, even on Sundays…”; “…I had an issue with my spine and had to teach classes from my bed, the posture and long hours sitting without being able to stand up…”; “…other colleagues experienced dry eye syndrome…”; “…I have a pillow on my back, a cushion for support, I adjust my workspace…”; “…I only delegate domestic tasks, maybe some academic tasks if they are mechanical…”; “…performing tasks for others causes stress, but I support them with affection.”	Physical and muscular pains.
S6F58	Female	“High work burden”; “I found it difficult to use technological tools like Zoom, Classroom.”	High workload.
S7M38	Male	“…there was an overload at the beginning of the pandemic…”; “…we felt compelled to seek help from others to solve problems, in some cases…”; “…in the academic aspect, I don’t usually do it…”; “…headaches and body pains arise when submitting work…”	Physical pains and mental exhaustion.
S8F59	Female	“It was a sudden and stressful change, very overloaded. When reviewing all the students’ theses, I felt very overwhelmed.”	High work overload.
S9F55	Female	“It was stressful, being glued to the computer, and research became more complicated due to spending many hours. This stressed both the teacher and the student.”	High work overload.
S10F55	Female	“During virtual attendance, work presentations were more dynamic, and the new way of evaluating students was not stressful.”	Easy adaptation.

**Figure 2.**
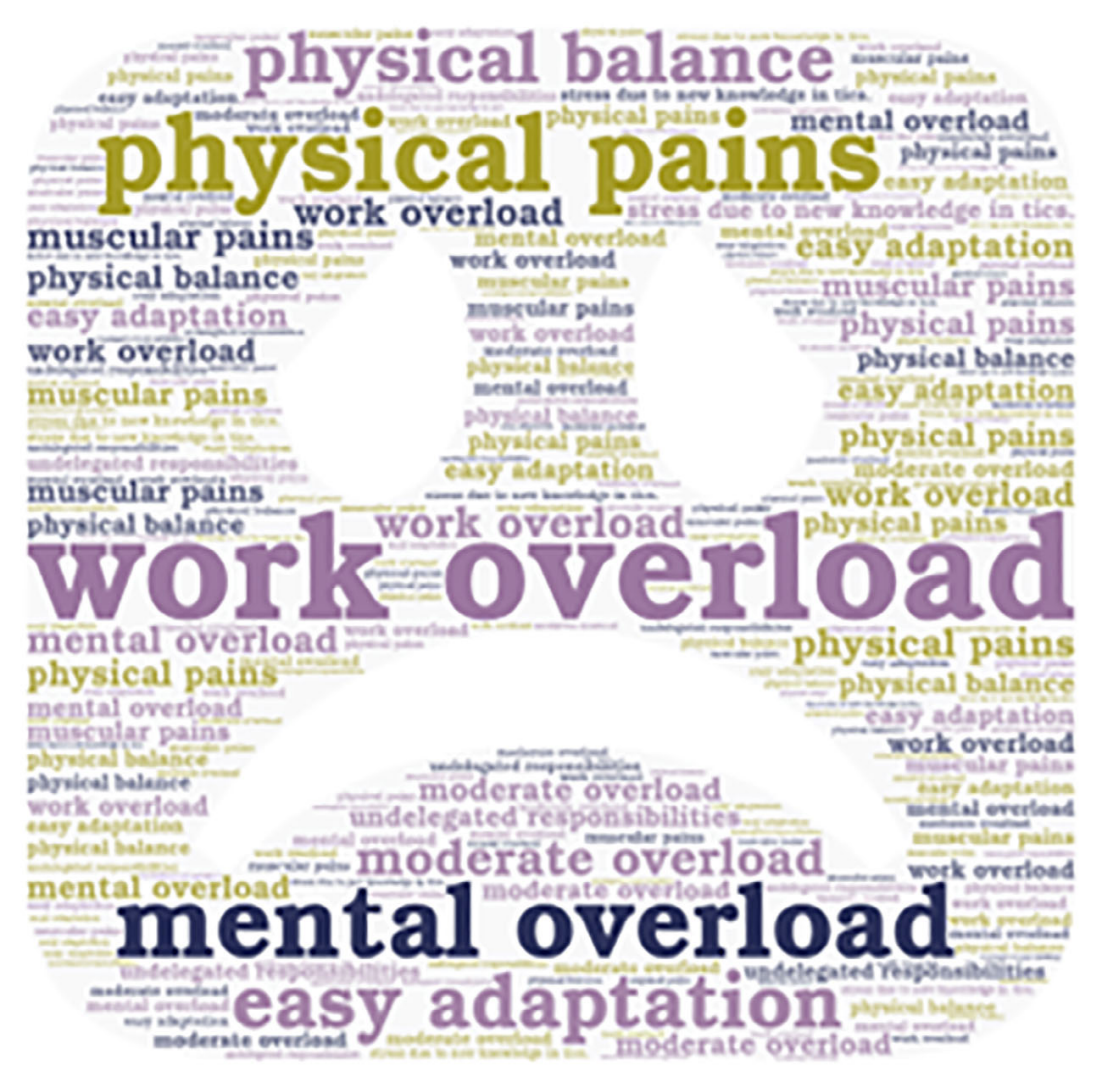
Emerging contents of the work overload subcategory.

**Table 5.** Responses of the interviewees on “Social and Emotional Interaction” subcategory.

Subject code	Gender	Social and Emotional Interaction	Emerging concepts
S1F55	Female	“…The best therapy is to work, when I am in the classroom, I forget about everything, or in any case, I talk to the students…”	Conexión con el estudiante es terapéutica.
S2F55	Female	“…When the student does not do the proposed activities, it produces uncertainty, doubt, fear, and restlessness regarding the authorship of their work…”	Ethical questioning.
S3M53	Male	“…A pleasant nature, we must know how to channel emotions…”; “if they are of joy, they can be shared…“; ”…not to convey annoyances to the students…”	Emotion channeling.
S4F58	Female	“…Take a deep breath, try to calm myself, and then enter the classroom. But if it is too severe, I share with the students that my mood might be affected due to adverse situations, to create a student-teacher bond, and request their understanding in such cases…”	Empathy and connection with the student.
S5F49	Female	“…I sing, talk, try not to show a bad face to the student…”; “…I tend to somatize with the colon and the digestive system…”; “…with my colleagues, we laugh, gossip, take a break during the day…”; “…dinner with my spouse, going for a walk, that helps…”	Recreational activities that foster connections with others.
S6F58	Female	“…I must keep my personal problems to myself…”; “I must give a hundred percent in everything…”; “…I enjoy walking, engaging in activities, seeking mental peace…”	Recreational activities for emotional channeling.
S7M38	Male	“…I try not to let it show in classes…”; “…I take deep breaths…”; “…there are moments of stress that I channel through physical exercise; one night, I went for a run at two in the morning…”	Physical activities for emotional channeling.
S8F59	Female	“…Whether sad or happy, I put on my best face…”; “…if I was feeling sad, I felt the need to be hugged…”; “…another way to release stress is by going out of the house, looking at things, not buying anything, but arriving home feeling calm…”	Channeling emotions and spending time alone.
S9F55	Female	“I haven’t attended the classroom when emotions overwhelm us; it’s better to be alone.”	Taking distance and channeling emotions.
S10F55	Female	“…Take a deep breath, wipe away the tears, and inform the students about my emotional state. Following a routine helps to forget about the problems…”; “…I walk by the beach, play salsa music, and it changes my emotions…”	Channeling emotions, recreational activities, and informing the student in advance.

**Figure 3.**
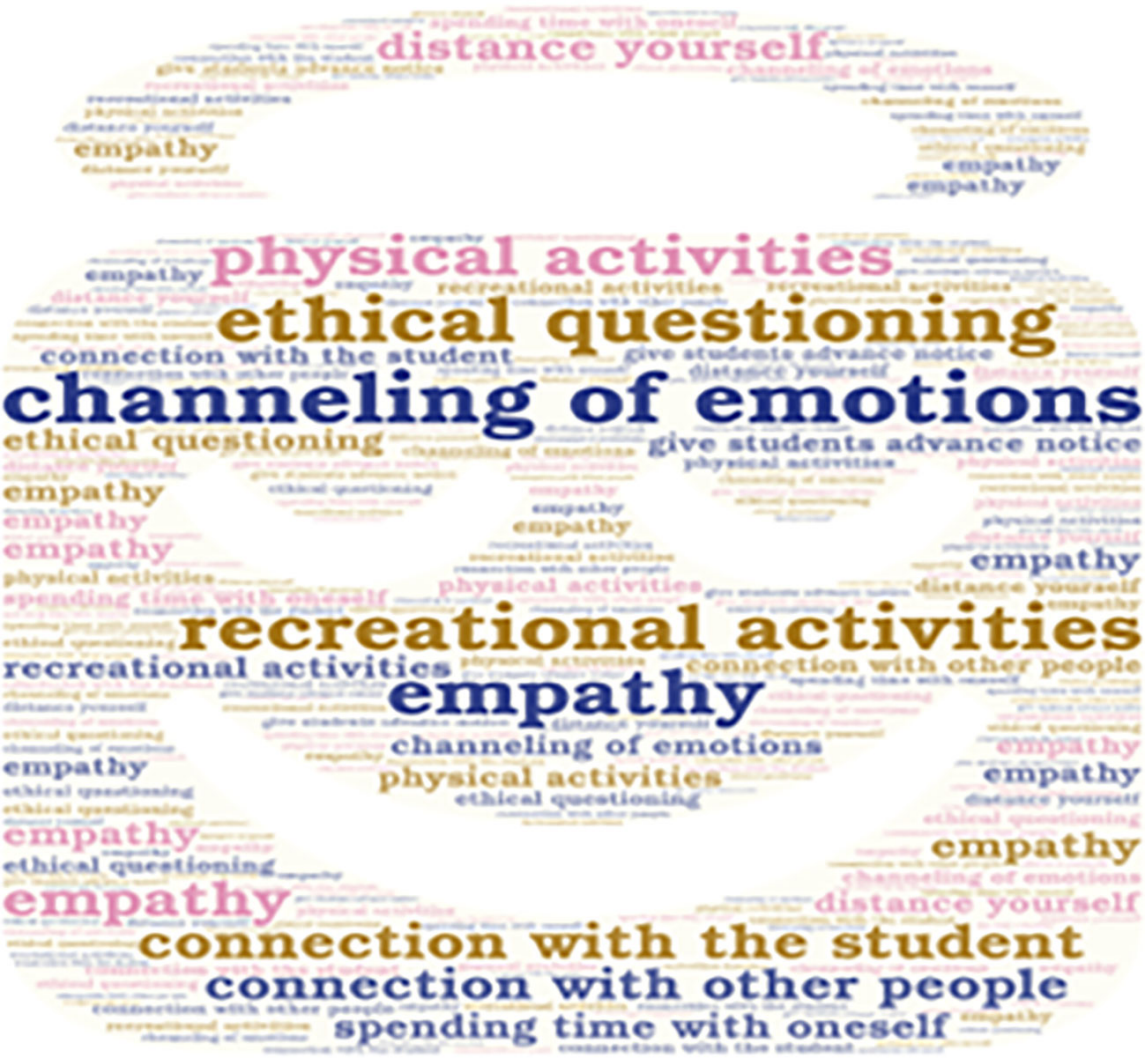
Emerging contents of the subcategory “Social and Emotional Interaction”.

**Table 6.** Responses of the interviewees on adaptation to new technologies.

Subject code	Gender	Adaptation to new technologies	Emerging concepts
S1F55	Female	“I conduct both synchronous and asynchronous classes, but I meet with them throughout the week to provide guidance. I use technology to upload materials, such as slides and resources that will be used in classes, and the rest is developed in person.”	Pragmatic adaptation.
S2F55	Female	“Technological teaching will be strengthened, which is why it is essential to be collaborators and work with technological tools. We must know how to choose…”	Positive adaptation.
S3M53	Male	“…I look for ways to achieve self-training, self-learning.”; “…it allows us to renew ourselves, and we are obligated to learn and apply them…”; “…I continue to use virtual platforms…”; “…there is nothing better than face-to-face teaching…”; “…The teacher must familiarize themselves with it and continue their education…”	Selective adaptation.
S4F58	Female	“…I knew about technological tools, but I didn’t use them.”; “…it is necessary to keep updating ourselves, like Chat GPT, AI, which students are using…”	Adaptation out of necessity.
S5F49	Female	“…I’m used to teaching in a virtual manner. I tell the student, ’Share your screen, erase that, change that, look for this,’ and they are more productive in the virtual environment…”; “…in face-to-face settings, a student brings me a manuscript, but it’s not productive…”; “…I miss technology; when I’m in a virtual setting, I interact more because there are more technological resources available. I can access databases, and the immediacy and speed at which learning develops are better…”; “…technologies will change perspectives. There will be things that cannot be replaced, such as situations where we can listen to students, touch them, look into their eyes; technology won’t be able to do that…”	Positive adaptation.
S6F58	Female	“…I haven’t gotten along very well with technology; it seemed complicated to me…”	Slow adaptation.
S7M38	Male	“…The teacher’s attitude is always of service…”; “…the physical encounter with the student is vital”; “…the teacher must provide a space to understand what is happening and address the needs.”	Slow adaptation.
S8F59	Female	“…We made several mistakes, but we had to learn from the fear…”; “…we must continue to train ourselves, trying to learn…”	Adaptation out of necessity.
S9F55	Female	“…I update myself out of necessity, and I have learned new technologies”; “…I have used programs like Canva, Cerebriti, and others.”	Positive adaptation.
S10F55	Female	“…I am not as skilled as the young people nowadays, but I can easily detect it at first glance because I’m familiar with Chat GPT and several AI technologies…”; “…one must know how to provide good input…”	Pragmatic adaptation.

**Figure 4.**
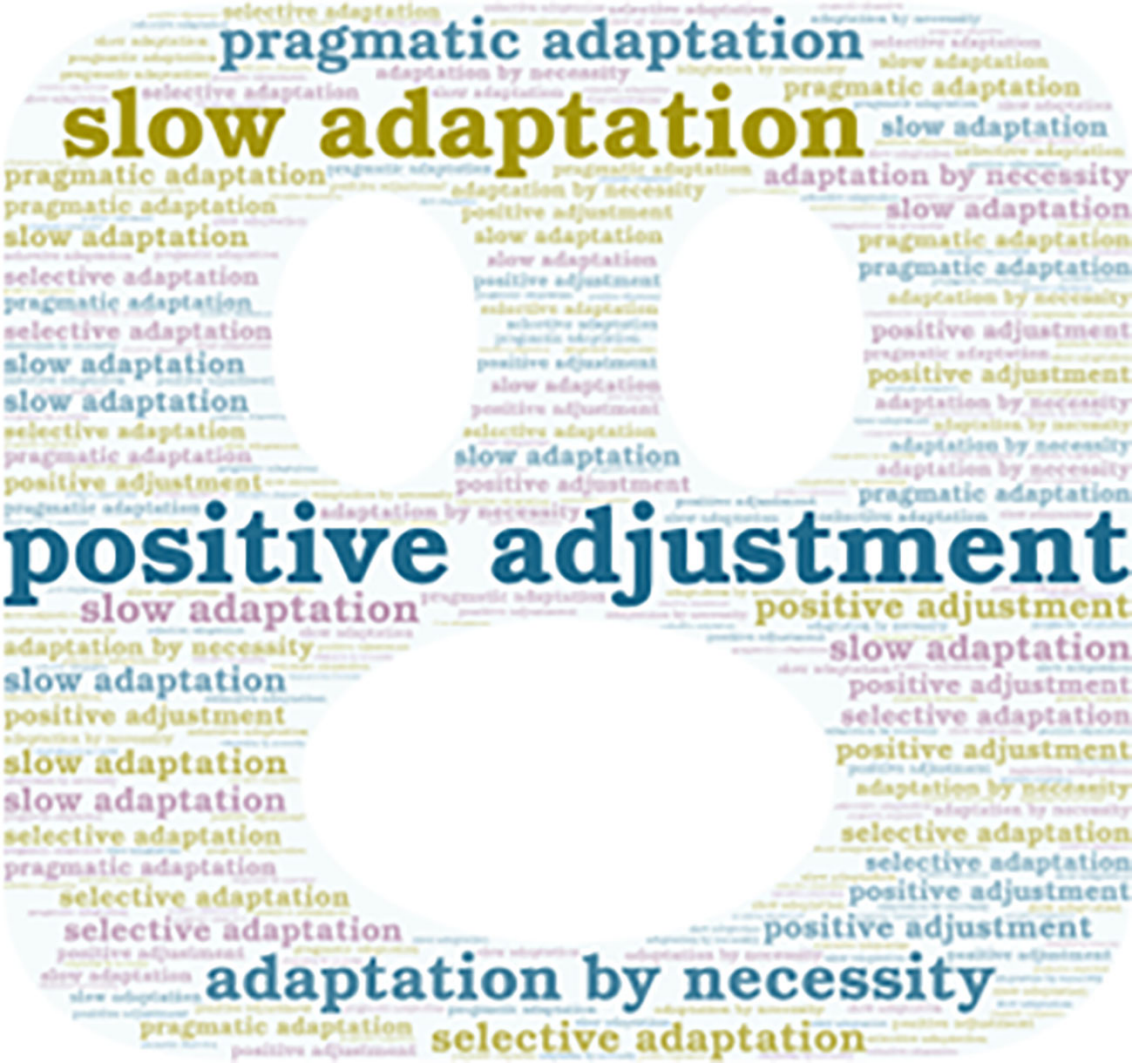
Emerging contents of the subcategory “Adaptation to New Technologies.”

**Figure 5.**
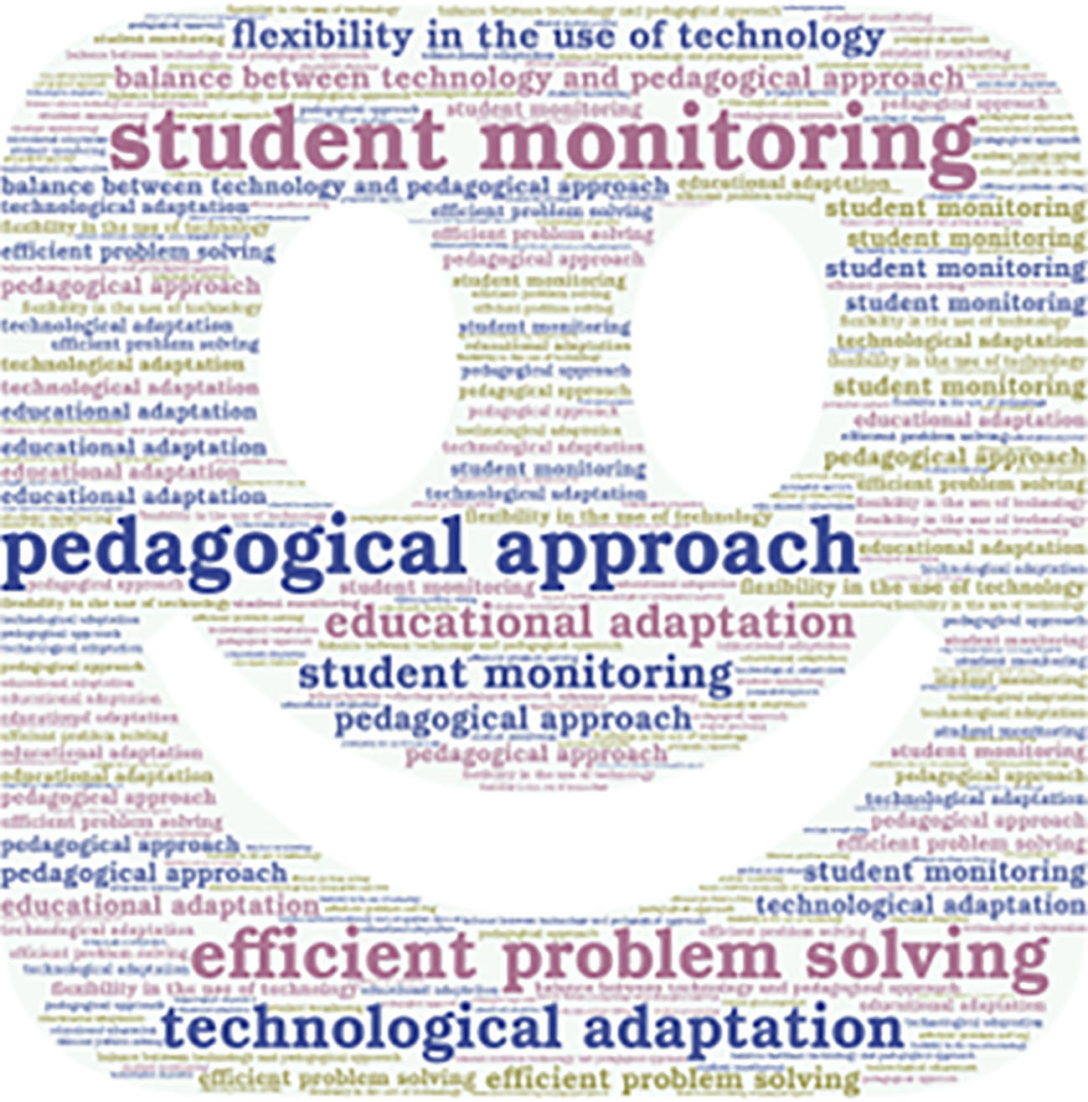
Emerging contents of the subcategory “expectations and quality of teaching.”

Additionally, it is important to mention that the emerging concepts are also presented in Figures 2, 3, 4, and 5.

## Results

The results were grouped according to the established subcategories.

Some interviewees consider work overload as moderate and high, which, in some cases, led to physical and emotional exhaustion. However, they are committed to providing quality education and rely on technological tools to optimize the teaching processes. University educators are facing various levels of work overload that can affect their physical and mental well-being. Although some can easily handle the changes.

Some interviewees emphasize the importance of emotional connection between teachers and students in order to avoid affecting the teaching process. There is also a need to channel emotions appropriately to carry out the teaching function. This can be achieved through recreational activities, physical activities, or spending time alone. However, one teacher prefers to distance themselves from others.

Some interviewees adapt to technology positively, meaning they find value and benefit in using it. Two interviewees expressed some difficulty in using technological tools, preferring traditional methods. Therefore, their adaptation is slow.

Others mentioned that they only use certain technological tools when they need them in their classes, which can be considered as pragmatic adaptation. Others utilize technology for specific functions related to their teaching subject, adapting to technology selectively. Finally, there are those who use certain tools because the institution requires them to do so, but it’s not a personal choice; therefore, their adaptation is out of necessity.

**Table 7.** Responses of the interviewees regarding expectations and quality of teaching.

Subject code	Gender	Expectations and quality of teaching	Emerging concepts
S1F55	Female	“…The university is a space that allows me to appreciate the students’ learning, but it also obliges me to train and stay up to date, as they are more tech-savvy and always have something new to share…”	Balance between technology and pedagogical approach.
S2F55	Female	“…To talk about the quality of teaching is to talk about the indicator of satisfaction; “…there are students in specializations who do not handle technologies or even their emails, there is a gap in the achievement of their autonomy…”	Technological adaptation and student monitoring.
S3M53	Male	“…There is excessive freedom for the student in education…”; “today’s students do not read, do not provide constructive criticism, and lack the appropriate commitment…”; “if I see effort from the student, then I must reinforce their learning…”; “…this has repercussions on future health due to the number of hours spent attending to the students…”	Flexibility in the use of technology and student monitoring.
S4F58	Female	“…Neither theoretical nor meticulous, they come with basic knowledge, and sometimes we leave them to their own devices, and they end up producing work without coherence…”; “…it is necessary for them to experiment and make mistakes so they can progress…”; “…it is essential to conduct a diagnosis on the first day of class…”	Efficient problem-solving and student monitoring.
S5F49	Female	“…I never tell the student that they are wrong…”; “…despite the fact that technologies may generate stress, they also provide a valuable resource for conducting research processes…”	Efficient resolution of technological problems and student monitoring.
S6F58	Female	“…I am a person who develops the theory and its particularities and leaves the practical part to the student, where they must conduct research. If they have any doubts about the class, I address their concerns…”; “…I rely on delegates to support those students who need more assistance…”; “…teaching will not change overnight; it is a slow process, and the change will be generational…”	Pedagogical approach and student monitoring.
S7M38	Male	“…I hated the type of teaching where the teacher just talks and talks and talks; it’s a very cold strategy…”; “…it’s relevant for the student to develop practical skills…”	Pedagogical adaptation.
S8F59	Female	“…I have to explain things meticulously, but I also need to combine different teaching methods and make myself understood. Sometimes I have doubts, wondering if they have really understood what I explained…”	Pedagogical approach and student monitoring.
S9F55	Female	“…Teachers who are content experts, meaning experts in a specific subject, designing a syllabus, for example, developing topics like artificial intelligence…”; “…I attend to students in a personalized manner…”	Technological adaptation and student monitoring.
S10F55	Female	“…I provide them with all the details, explaining how they should present their assignments…”; “…I use a colloquial and friendly language in individual sessions…”	Pedagogical approach.

The interviewed teachers are constantly updating themselves. However, their main focus is on monitoring the students, who face difficulties in acquiring knowledge. Furthermore, some interviewed professionals efficiently solve academic problems, adapting to the circumstances, and in certain cases, making use of technology.

## Discussion

Upon analysing the responses of the interviewees regarding their experiences and perceptions of techno-stress, new subcategories emerge for analysis.

### Work overload

Several studies have delved into the educational context, uncovering disparities in the findings. Some indicate high levels ( Alvites, 2019), while others point to moderate levels ( Rivadeneira Guerrero *et al.*, 2020; Zheng *et al.*, 2022).

Work overload significantly impacts the physical and psychological well-being of teachers, extending beyond just a heavy workload. Teachers experience physical and mental exhaustion, leading to elevated levels of stress and anxiety ( Alvites, 2019; Rivadeneira Guerrero *et al.*, 2020). Work overload manifests in various ways, from planning class sessions to grading assignments and assessments. However, despite these challenges, teachers demonstrate commitment, striving to provide quality education.

Upon analysing the results, it becomes evident that work overload should not be normalized or overlooked. Therefore, it is essential for those responsible for establishing educational policies to implement necessary measures for the well-being of teachers, fostering a healthy environment.

The research team suggests further exploration of the long-term consequences of techno-stress, not only for teachers but also for students. The aim is to develop strategies that promote the well-being of teachers.

### Social and emotional interaction

Teachers are capable of effectively managing their emotions through various strategies. The most commonly used strategies include engaging in physical activities and communicating with their students ( Romero-Martín & Fraile-Aranda, 2017). Promoting effective communication creates an environment conducive to understanding and empathy. Through these mentioned strategies, teachers maintain emotional balance, enabling them to carry out their pedagogical work despite emotional challenges or difficulties that may arise ( Genimon Vadakkemulanjanal *et al.*, 2021). However, recognizing emotions, being inherent to human nature, can be difficult ( Francisti *et al.*, 2023). Yet, an educator who manages their emotions and establishes a meaningful connection with their students will cultivate a relationship built on trust and respect.

Appropriate emotional management by teachers can have an impact on the learning process. If students perceive an emotionally healthy environment, they are more likely to be motivated to participate in class activities with a positive attitude toward learning. These findings align with the proposed vital components of teaching ( Ch’ng, 2019; Drewelow, 2020; Wetcho & Na-Songkhla, 2022).

When addressing the theme of social and emotional interaction from a scientific perspective, the importance of emotional management needs to be grounded. This applies not only to the educational sphere but to any context where individuals interact with others.

Following the respective analysis, the team suggests further exploration of this subcategory to identify the effectiveness of different emotional management strategies, such as physical activities and effective communication, in comparison with other techniques like meditation, socioemotional skill training, or psychological counselling. Research could be conducted to examine the impact on the emotional well-being of teachers or to track teachers participating in training programs. This could involve creating both control and experimental groups and subsequently comparing short-term or long-term results. It is imperative to provide robust findings to establish educational policies that promote the emotional well-being of the educational community.

### Adaptation to new technologies

The interviewees expressed that, despite possessing technological skills, they remain willing to continue updating themselves, even if this leads to pressure or stress for them ( Alvites, 2019; Rivadeneira Guerrero *et al.*, 2020). However, it has also been observed that some teachers opt to stick with traditional practices, attempting to distance themselves from technology, or their use of technology is less frequent compared to their colleagues. This diversity of attitudes among teachers towards technology reveals that some leverage the opportunities technology offers and are open to adapting to changes ( Bendito Cañizares & Sánchez Botas, 2021; Pribeanu & Gabriel Gorghiu, 2022; Shulga *et al.*, 2021). On the other hand, there are teachers who prefer to remain in a comfortable space and use technology only when necessary or not use it at all ( Abarca *et al.*, 2022; De Lima Santana *et al.*, 2022; Morales-Rodríguez, 2021).

Following these findings, a deeper exploration of the reasons behind attitudes towards technology in the academic context is necessary. Additionally, identifying factors that influence teachers to either embrace or resist the adoption of new technological trends is essential. By understanding the results of this exploration, strategies can be designed and implemented in the form of updates or workshops within the academic institution.

### Expectations and quality of teaching

It is evident that technology is present in the daily activities of both teachers and students. However, for the optimal use of technology, the active participation of teachers through concrete actions is required ( Abarca *et al.*, 2022). Technology alone does not guarantee quality teaching. Therefore, it is essential for teachers to be capable of utilizing technological resources to provide guidance and deliver information to students through various electronic mediums ( Delgado-García *et al.*, 2017). The relationship between technology and the willingness of both teachers and students is crucial in enhancing the quality of education.

Investigating the utilization of technology and its impact on learning, as well as identifying challenges faced in its implementation, is of paramount importance. The findings from such research can be harnessed to design effective strategies and promote the integration of technology within the teaching process. This way, the potential that technology offers can be fully leveraged.

### Contributions and limitations of the study

The research provides insights and tools that can be utilized by teachers, students, or anyone who might be using technology inappropriately. The findings related to techno-stress serve as reference points for enhancing the quality of teaching and promoting efficient technology use within the teaching-learning process. A deeper understanding of the relationship among students, teachers, and technology contributes to emotional well-being for the educational community.

The research did have several limitations, such as the challenge of objectively measuring techno-stress. The findings are based on teachers’ perceptions, and these perceptions vary among interviewees. Therefore, a qualitative research approach captures the lived experiences of each participant. Another limitation was the influence of personal factors related to technology. For instance, a participant experienced with technology might not recognize that they are actually dealing with technological dependency when interviewed about techno-stress. It is suggested that further research on techno-stress takes an integral and multidisciplinary approach due to the complexity of the phenomenon, whether it serves as a cause or consequence of excessive technology use.

## Data Availability

Zenodo. Raw data for: Navigating techno-stress: A qualitative exploration of university faculty’s experiences and perspectives in the Peruvian context amidst the return to classes and the post-COVID-19 era. DOI: https://doi.org/10.5281/zenodo.8384995 ( Suyo-Vega *et al., *2023). This project contains the following data:
- Raw data for: Navigating techno-stress: A qualitative exploration of university faculty’s experiences and perspectives in the Peruvian context amidst the return to classes and the post-COVID-19 era Data are available under the terms of the Creative Commons Attribution 4.0 International license (CC-BY 4.0). Zenodo SRQR checklist. DOI: https://doi.org/10.5281/zenodo.8264791

## References

[ref1] Abarca R Buenaño C Mejía F : La pandemia COVID-19 inductor de tecnoestrés en docentes de la educación ecuatoriana de segundo nivel. *Boletín de Malariología y Salud Ambiental.* 2022;62(2):266–279. 10.52808/bmsa.7e6.622.018

[ref2] Alvites G : Estrés docente y factores psicosociales en docentes de Latinoamérica, Norteamérica y Europa. *Journal of Indo - European Studies.* 2019;47(3/4):141–159.

[ref3] Bendito Cañizares MT Sánchez Botas FJ : Changes in the teaching methodology and the study of the adaptation of students to the new technology: From the canvas methodology to the accentuation of the flipped methodology. *International Conference Mobile Learning 2021, ML 2021.* 2021;150–160. 10.33965/ml_icedutech2021_202102l019

[ref45] Castellanos-Alvarenga LM Miranda-Rosas LF Quiroz-Moya MS : Emotional regulation and technostress in higher education teachers. A systematic review. *Rev. Logos Cienc. Tecnol.* 2024;16:193–212. 10.22335/rlct.v16i1.1878

[ref4] CEPAL: La educación en tiempos de la pandemia de Covid-19. *Unesco.* 2020. 10.1097/ACM.0000000000004003

[ref5] Ch’ng LK : Learning emotions in E-learning: How do adult learners feel? *Asian Journal of Distance Education.* 2019;14(1):34–46.

[ref6] Chávez Irigoyen C Penelas Ronso Merino E : Brechas de género en la gobernanza universitaria y la carrera docente en el Perú. *Revista Educación Superior y Sociedad (ESS).* 2021;33(2):738–766. 10.54674/ess.v33i2.346

[ref7] Corrente M Ferguson K Bourgeault IL : Mental Health Experiences of Teachers: A Scoping Review. *Journal of Teaching and Learning.* 2022;16(1):23–43. 10.22329/jtl.v16i1.6856

[ref8] De la Espriella R Gómez Restrepo C : Teoría Fundamentada. *Revista Colombiana de Psiquiatria.* 2020;49(2):127–133. 10.1016/j.rcp.2018.08.002 32446420

[ref9] De Lima Santana L Hainiski Ramos T De Biagi Ziesemer N : Fatores intervenientes na qualidade de vida docente durante a pandemia da COVID-19. *Revista Actualidades Investigativas En Educación.* 2022;22(1):1–32. 10.15517/aie.v22i1.447441

[ref10] Decataldo A Fiore B : Digital-insecurity and overload: The role of technostress in lecturers’ work-family balance. *Italian Journal of Sociology of Education.* 2022;14(3):75–102. 10.14658/pupj-ijse-2022-3-4

[ref11] Delgado-García M García-Prieto FJ Gómez Hurtado I : Moodle y Facebook como herramientas virtuales didácticas de mediación de aprendizajes: opinión de profesores y alumnos universitarios. *Revista Complutense de Educación.* 2017;29(3):807–827. 10.5209/rced.53968

[ref46] Díaz-Bravo L Torruco-García U Martínez-Hernández M : La entrevista, recurso flexible y dinámico. *Investigación En Educación Médica.* 2013;2:162–167. 10.1016/S2007-5057(13)72706-6

[ref12] Drewelow I : A positive psychology perspective on designing a technology-mediated learning experience: Engagement and personal development. *Journal for the Psychology of Language Learning.* 2020;2(1):90–115. 10.52598/jpll/2/1/5

[ref13] Estrada Araoz GE Gallegos Ramos NA Huaypar Loayza KH : Tecnoestrés en estudiantes de una universidad pública de la Amazonía peruana durante la pandemia COVID-19. *Revista Brasileira De Educação Do Campo.* 2021;6(e12777):1–19. 10.20873/uft.rbec.e12777

[ref14] Fernández Villacres GE Viscaino Naranjo FA Llerena Ocaña LA : Determinación de la fatiga ocular debido a teletrabajo en los docentes de la universidad UNIANDES de Ecuador. *Revista Dilemas Contemporáeos: Educación, Política y Valores.* 2021 March;1–19. 10.46377/dilemas.v8i3.2673

[ref15] Francisti J Balogh Z Turčáni M : The use of internet of things technology in the pedagogical process. 2023. 10.33225/balticste/2023.65

[ref16] Garcés-Delgado Y García-Álvarez E López-Aguilar D : Incidencia del género en el estrés laboral y burnout del profesorado universitario. *REICE. Revista Iberoamericana Sobre Calidad, Eficacia y Cambio En Educación.* 2023;21(3):41–60. 10.15366/reice2023.21.3.003

[ref17] Genimon Vadakkemulanjanal J Kennedy A Nero A : Impact of technology readiness and techno stress on teacher engagement in higher secondary schools. *Digital Education Review.* 2021;40:51–65. 10.1344/der.2021.40.51-65

[ref18] Guba EG Lincoln YS : Competencia de paradigmas en la Investigación cualitativa. *Paradigmas en competencia en la investigación cualitativa.* 1994;105–117.

[ref19] Heidari S Babor TF De Castro P : Sex and Gender Equity in Research: rationale for the SAGER guidelines and recommended use. *Research Integrity and Peer Review.* 2016;1(1):2. 10.1186/s41073-016-0007-6 29451543 PMC5793986

[ref20] Hernández-Sampieri R Mendoza Torres CP : *Metodología de la investigación. Las rutas cuantitativa, cualitativa y mixta.* McGraw-Hill;2018.

[ref21] Jimenez EC : Impact of mental health and stress level of teachers to learning resource development. *Shanlax International Journal of Education.* 2021;9(2):1–11. 10.34293/education.v9i2.3702

[ref22] Kaya H : Investigation of the effect of online education on eye health in Covid-19 pandemic. *International Journal of Assessment Tools in Education.* 2020;7(3):488–496. 10.21449/ijate.788078

[ref47] Khlaif ZN Khalili F Affouneh S : How remote leaning during crisis affect technostress levels experienced by academicians. *Education and Information Technologies.* 2023a;28:1–26. 10.1007/s10639-023-11651-6 36818433 PMC9924893

[ref48] Khlaif ZN Sanmugam M Ayyoub A : Impact of technostress on continuance intentions to use mobile technology. *Asia-Pacific Education Researcher.* 2023b;32:151–162. 10.1007/s40299-021-00638-x

[ref49] Khlaif ZN Sanmugam M Joma AI : *Factors influencing teacher’s Technostress experienced in using emerging technology: A qualitative study.* Netherlands: Springer;2023c. 10.1007/s10758-022-09607-9

[ref23] Korstjens I Moser A : Series: Practical guidance to qualitative research. Part 4: Trustworthiness and publishing. *European Journal of General Practice.* 2018;24(1):120–124. 10.1080/13814788.2017.1375092 29202616 PMC8816392

[ref24] Manzur-Vera G Rodriguez C Vargas M : Reflexiones de pandemia en la educación superior: revisión teórica del estrés laboral en el rol docente. *Technological Innovations Journal.* 2022;1(3):60–75. 10.35622/j.ti.2022.03.004

[ref25] Maslach C Schaufeli WB Leiter MP : Job Burnout. *Annu. Rev. Psychol.* 2001;52:397–422. 10.1146/annurev.psych.52.1.397 11148311

[ref26] Mena Freire MA Ruiz Olarte AM Vargas Espín A d P : Diferencias de género en la percepción de estrés en universitarios del Ecuador. *Ciencia Latina Revista Científica Multidisciplinar.* 2022;6(6):5916–5928. 10.37811/cl_rcm.v6i6.3850

[ref27] Morales-Rodríguez FM : Fear, stress, resilience and coping strategies during covid-19 in Spanish university students. *Sustainability (Switzerland).* 2021;13(11). 10.3390/su13115824

[ref28] Nieto Montesinos RM Paredes Zavala JM Rivera Flores GW : Sumajg Warmi: Situación, retos y oportunidades de mujeres científicas en la Costa, Sierra y Selva del Perú. *CIES.* 2019.

[ref29] Pansini M Buonomo I De Vincenzi C : Positioning Technostress in the JD-R Model Perspective: A Systematic Literature Review. *Healthcare (Switzerland).* 2023;11(3). 10.3390/healthcare11030446 36767021 PMC9914396

[ref30] Pribeanu C Gabriel Gorghiu E-AS : Drivers of continuance intention to use the online learning platform after the Covid-19 pandemic. *Problems of Education in the 21st Century.* 2022;80(5):724–736. 10.33225/pec/22.80.724

[ref31] Rakhimgalieva P Serikbayeva N Seiitkazy P : Adaptation of students to professional activity through innovative technologies. *World Journal on Educational Technology: Current Issues.* 2021;13(4):1102–1123. 10.18844/wjet.v13i4.6312

[ref32] Rivadeneira Guerrero MF Sola Villena JH Chuquimarca Mosquera MC : Experiencia y resultados de un proceso educativo interdisciplinario para la promoción de salud en universitarios. *Hacia La Promoción de La Salud.* 2020;25(2):109–123. 10.17151/hpsal.2020.25.2.12

[ref33] Romero-Martín R Fraile-Aranda A : Evaluación formativa, competencias comunicativas y TIC en la formación del profesorado. *Comunicar.* 2017;25(52):73–82. 10.3916/C52-2017-07

[ref34] Sánchez-Macías A Flores-Rueda IC Veytia-Bucheli MG : Tecnoestrés y adicción a las tecnologías de la información y las comunicaciones (TIC) en universitarios mexicanos: diagnóstico y validación de instrumento. *Formación Universitaria.* 2021;14(4):123–132. 10.4067/s0718-50062021000400123

[ref35] Shulga TI Li YJ Krokhina JA : Digital technologies’ impacts on student social adaptation during Coronavirus pandemic. *World Journal on Educational Technology: Current Issues.* 2021;13(4):740–748. 10.18844/wjet.v13i4.6261

[ref36] Simona G Hana M Lenka D : Ciberostracismo: Consecuencias emocionales y conductuales en las interacciones en redes sociales. *Comunicar.* 2021;67:9–20.

[ref37] Suyo-Vega JA Meneses-La-Riva ME Fernández-Bedoya VH : Mental Health Projects for University Students: A Systematic Review of the Scientific Literature Available in Portuguese, English, and Spanish. *Frontiers in Sociology.* 2022;7. 10.3389/fsoc.2022.922017 35898816 PMC9309378

[ref52] Suyo-Vega JA Meneses-La-Riva ME Fernández-Bedoya VH : Raw data for: Navigating techno-stress: A qualitative exploration of university faculty’s experiences and perspectives in the Peruvian context amidst the return to classes and the post-COVID-19 era. *Zenodo.* 2023. 10.5281/zenodo.8384995

[ref38] Tondeur J Hermans R Braak J van : Exploring the link between teachers’ educational belief profiles and different types of computer use in the classroom. *Computers in Human Behavior.* 2008;24(6):2541–2553. 10.1016/j.chb.2008.02.020

[ref39] Torres-Hernández EF : Vocation and burnout in Mexican teachers. *Educacion XX1.* 2023;26(1):327–346. 10.5944/educxx1.32954

[ref40] UNESCO - IESALC: Mujeres en la educación superior: ¿la ventaja femenina ha puesto fin a las desigualdades de género? *Organizacion de Las Naciones Unidas Para La Educacion, Ciencia y La Cultura.* 2021;1(7):1–68.

[ref41] Vives Varela T Hamui Sutton L : La codificación y categorización en la teoría fundamentada, un método para el análisis de los datos cualitativos. *Investigación En Educación Médica.* 2021;10:97–104. 10.22201/fm.20075057e.2021.40.21367

[ref50] Wang C Lee MKO Yang C : Understanding problematic smartphone use and its characteristics: A perspective on behavioral addiction. *Lecture Notes in Information Systems and Organisation.* 2016;17:V–VI. 10.1007/978-3-319-30133-4

[ref51] Wang K Shu Q Tu Q : Technostress under different organizational environments: An empirical investigation. *Computers in Human Behavior.* 2008;24:3002–3013. 10.1016/j.chb.2008.05.007

[ref42] Wetcho S Na-Songkhla J : An investigation of pre-service teachers using mobile and wearable devices for emotion recognition and social sharing of emotion to support emotion regulation in mCSCL environments. *Contemporary Educational Technology.* 2022;14(2):1–15. 10.30935/cedtech/11668

[ref43] Zheng M Asif M Tufail MS : COVID academic pandemic: Techno stress faced by teaching staff for online academic activities. *Frontiers in Psychology.* 2022;13(July):1–6. 10.3389/fpsyg.2022.895371 35992455 PMC9384887

[ref44] Zotova M Likhouzova T Shegai L : The use of moocs in online engineering education. *International Journal of Engineering Pedagogy.* 2021;11(3):157–173. 10.3991/IJEP.V11I3.20411

