# Supplementary material for: Adapting to a New Normal: Peruvian University Faculty’s Experiences with Techno-Stress Post-COVID-19
Source: F1000Res. 2024 Nov 11;12:1381. Originally published 2023 Oct 20. [Version 3] doi: 10.12688/f1000research.141432.3 (PMC11574330; doi:10.12688/f1000research.141432.3)
Supplement: Peer review history

# Reviewer report: Reviewer response for version 3

**DOI:** 10.5256/f1000research.174065.r339679

**Published:** 2024-11-18

**Authors:** Rajalakshmi S.A.

**Affiliations:** 1 Department of Social Work, Amrita School of Social and Behavioural Sciences, Amrita Vishwa Vidhyapeetham, Coimbatore, Tamil Nadu, India

I am pleased with the changes made by the authors in their revised manuscript. The reformulation of the title enhances its appeal and ensures clear identification in databases. The revised abstract provides greater clarity by specifying the qualitative methodology and incorporating additional elements. The inclusion of the "Literature Review" subsection effectively delineates Maslach's Burnout Theory from Tondeur’s work, adding depth to the introduction. Table 1 is a valuable addition, presenting the 12 questions linked to each research subcategory concisely. The enhancements in the recommendations and limitations section, particularly the emphasis on the value of grounded theory in strengthening findings and guiding future research and policymaking, are commendable. The overall improvements in writing further enhance the article's quality.

Is the work clearly and accurately presented and does it cite the current literature?

Yes

If applicable, is the statistical analysis and its interpretation appropriate?

Not applicable

Are all the source data underlying the results available to ensure full reproducibility?

Yes

Is the study design appropriate and is the work technically sound?

Yes

Are the conclusions drawn adequately supported by the results?

Yes

Are sufficient details of methods and analysis provided to allow replication by others?

Partly

Reviewer Expertise:

Social Psychology, Positive Psychology, Education

I confirm that I have read this submission and believe that I have an appropriate level of expertise to confirm that it is of an acceptable scientific standard.

# Reviewer report: Reviewer response for version 2

**DOI:** 10.5256/f1000research.171124.r328519

**Published:** 2024-10-08

**Authors:** Rajalakshmi S.A.

**Affiliations:** 1 Department of Social Work, Amrita School of Social and Behavioural Sciences, Amrita Vishwa Vidhyapeetham, Coimbatore, Tamil Nadu, India

The manuscript requires further revision as the suggested changes have not been incorporated. The title remains unrefined, and the abstract still lacks important details about the research method and design. Additionally, the references mentioned in the text have not been added to the reference list, and the interview questions have not been attached. The recommendations to enhance the results, discussion, and conclusion sections have also not been addressed. These revisions are important for improving the overall clarity and strength of the manuscript.

Is the work clearly and accurately presented and does it cite the current literature?

Yes

If applicable, is the statistical analysis and its interpretation appropriate?

Not applicable

Are all the source data underlying the results available to ensure full reproducibility?

Yes

Is the study design appropriate and is the work technically sound?

Yes

Are the conclusions drawn adequately supported by the results?

Yes

Are sufficient details of methods and analysis provided to allow replication by others?

Partly

Reviewer Expertise:

Social Psychology, Positive Psychology, Education

I confirm that I have read this submission and believe that I have an appropriate level of expertise to confirm that it is of an acceptable scientific standard, however I have significant reservations, as outlined above.

# Response

**Published:** 2024-10-26

**Authors:** VICTOR HUGO FERNANDEZ BEDOYA

**Affiliations:** Universidad Cesar Vallejo, Trujillo, La Libertad, Peru

- We have reformulated the research title to make it more appealing to the reader while maintaining the key terms for quick identification in databases.
- The abstract has been revised in the methodology section to specify that it is qualitative using grounded theory, and additional elements have been incorporated and reformulated.
- We have included a new subsection in the introduction titled "Literature Review," where we provide a brief introduction to separate Maslach's Burnout Theory from Tondeur’s work.
- Table 1 presents the 12 questions linked to each research subcategory.
- In the recommendations and limitations section, we have added content related to the use of grounded theory in this type of study and how it strengthens the findings, recommending its use to other researchers for future discussions and to policymakers for extracting the identified advancements.
- We have made some improvements in the writing.

# Reviewer report: Reviewer response for version 1

**DOI:** 10.5256/f1000research.154876.r234914

**Published:** 2024-03-09

**Authors:** Maria Amelia Eliseo

**Affiliations:** 1 Universidade Presbiteriana Mackenzie, São Paulo, State of São Paulo, Brazil

Dear Authors,

 The article presents a qualitative research carried out with 10 Higher Education teachers involving four aspects: Work Overload; Social and Emotional Interaction; Adaptation to new Technologies and Expectations and quality of Teaching. 

 It is a current and relevant subject in the area of Education.

 Here are some comments to improve the text.

- Although the title refers to the post COVID-19 era, it is not clear in the text this relationship. Perhaps it could only contextualize the COVID-19 era, with the obligation for higher education teachers to adhere to the use of technology for their online classes and treat techno-stress as a current reality in the academic community.
- Although the Introduction presents the theoretical foundation and the research question, it failed to show the contextualization of the problem and detail the research objectives.
- In Methods, you could detail in which period the interviews were conducted. How were they planned and conducted?
- How were the SAGER guidelines applied? 
- In Results, Figures 2, 3, 4, and 5 were not contextualized. What would the authors like to show to readers with these figures?
- At the end of the article I missed the conclusion and the notes of some future work.
- I suggest the following reference to support the discussions:[1]

Is the work clearly and accurately presented and does it cite the current literature?

Yes

If applicable, is the statistical analysis and its interpretation appropriate?

Partly

Are all the source data underlying the results available to ensure full reproducibility?

Yes

Is the study design appropriate and is the work technically sound?

Partly

Are the conclusions drawn adequately supported by the results?

Partly

Are sufficient details of methods and analysis provided to allow replication by others?

Partly

Reviewer Expertise:

She is a researcher at the research group TecInt - Interactive Technologies and MackLEAPS - Laboratory for Studies of Software Production Environments. Coordinates the research projects "ICT evaluation to boost the technological transformation of micro and small enterprises in the incorporation of Industry 4.0 with a focus on human factors" and "Usability and Accessibility Analysis in Inclusive Games for Seniors". He has experience in Human-Computer Interaction, Web Technologies (Accessibility and Usability), Virtual and Augmented Reality, Interaction Design.

I confirm that I have read this submission and believe that I have an appropriate level of expertise to confirm that it is of an acceptable scientific standard, however I have significant reservations, as outlined above.

## References

[1] : Navigating techno-stress: A qualitative exploration of university faculty's experiences and perspectives in the Peruvian context amidst the return to classes and the post-COVID-19 era. *F1000Research* .2023;12: 10.12688/f1000research.141432.1 10.12688/f1000research.141432.1 37476505

# Reviewer report: Reviewer response for version 1

**DOI:** 10.5256/f1000research.154876.r231297

**Published:** 2023-12-21

**Authors:** Zuheir N Khlaif

**Affiliations:** 1 Department of Humanities and Educational Sciences, An-Najah National University, Nablus, Palestinian Territory

Dear Authors,

 Thank you for submitting your manuscript for consideration by our journal. The topic is intriguing, and I appreciate the effort. However, there are several areas that need improvement before it can be considered for publication.

**Abstract:** Please include the purpose of your research, the context, the number of participants, and details about the methodology and data analysis process. While some of these elements are present, they are not sufficiently detailed.

**Introduction:** This section should clearly define your research problem. I was searching for the problem/motivation behind your research but did not find it. Additionally, the purpose of your study and its contribution to the field should be articulated.

 Literature review

 Improve your LR by adding new articles and related ones. I suggest the following articles to include in your research in different stages, especially the methodology  and discussion

 https://link.springer.com/article/10.1007/s40299-021-00638-x

 https://www.cell.com/heliyon/pdf/S2405-8440(23)06277-1.pdf

 https://www.mdpi.com/2227-9032/11/3/446

 https://link.springer.com/article/10.1007/s10758-022-09607-9

 https://link.springer.com/article/10.1007/s10639-023-11651-6

Is the work clearly and accurately presented and does it cite the current literature?

Partly

If applicable, is the statistical analysis and its interpretation appropriate?

Not applicable

Are all the source data underlying the results available to ensure full reproducibility?

Partly

Is the study design appropriate and is the work technically sound?

Partly

Are the conclusions drawn adequately supported by the results?

Partly

Are sufficient details of methods and analysis provided to allow replication by others?

No

Reviewer Expertise:

Technology integration

I confirm that I have read this submission and believe that I have an appropriate level of expertise to confirm that it is of an acceptable scientific standard, however I have significant reservations, as outlined above.

## References

[1] : Impact of Technostress on Continuance Intentions to Use Mobile Technology. *The Asia-Pacific Education Researcher* .2022; 10.1007/s40299-021-00638-x 10.1007/s40299-021-00638-x

[2] : Mobile technology features and technostress in mandatory online teaching during the COVID-19 crisis. *Heliyon* .2023;9(8) : 10.1016/j.heliyon.2023.e19069 e19069 10.1016/j.heliyon.2023.e19069 37636397 PMC10448022

[3] : Positioning Technostress in the JD-R Model Perspective: A Systematic Literature Review. *Healthcare (Basel)* .2023;11(3) : 10.3390/healthcare11030446 10.3390/healthcare11030446 36767021 PMC9914396

[4] : Factors Influencing Teacher’s Technostress Experienced in Using Emerging Technology: A Qualitative Study. *Technology, Knowledge and Learning* .2023;28(2) : 10.1007/s10758-022-09607-9 865-899 10.1007/s10758-022-09607-9

[5] : How remote leaning during crisis affect technostress levels experienced by academicians. *Educ Inf Technol (Dordr)* .2023; 10.1007/s10639-023-11651-6 1-26 10.1007/s10639-023-11651-6 36818433 PMC9924893

# Reviewer report: Reviewer response for version 1

**DOI:** 10.5256/f1000research.154876.r218114

**Published:** 2023-11-10

**Authors:** Rajalakshmi S.A.

**Affiliations:** 1 Department of Social Work, Amrita School of Social and Behavioural Sciences, Amrita Vishwa Vidhyapeetham, Coimbatore, Tamil Nadu, India

In order to enhance the quality of the article, the authors are encouraged to consider the following recommendations:

**1. Title:** The title could be refined for increased catchiness without compromising the essence of the research.

**2. Abstract:** It is suggested to augment the abstract by incorporating details about the research approach and design employed, providing a more comprehensive overview.

**3. Introduction:**

- Separate Maslach's Burnout theory and Tondeur’s work into distinct sections, designating Maslach's Burnout theory as the conceptual framework.
- Integrate relevant points from pioneer studies such as Wang et al. (2023) and Khlaif et al. (2023) to enrich the theoretical foundation.

**4. Methods:**

- If the researchers utilized unstructured interview methods, explicitly mention this in the design to preemptively address potential inquiries.
- If alternative interview methods were employed, consider presenting the questions to enhance transparency.

**5. Results:**

- Group emerging concepts and create a thematic map or figure with appropriate headings and captions to visually represent the findings effectively.
- Instead of a tabular format, consider presenting interviewee responses in narrative paragraphs, offering profound insights into their perspectives.
- Integrate information on emerging concepts directly into the results section to reinforce the direct link between results and discussion.

**6. Discussion:**

- Provide a more in-depth explanation of how grounded theory was employed in the research.
- Elaborate on the implications of adhering to SAGER guidelines and elucidate on the macro view derived from the results.
- it is advisable for the authors to adopt a more expansive perspective, incorporating insights from the results to enrich the discourse.

**7. Conclusion:**

- Craft a concise yet comprehensive conclusion that encapsulates the key findings and their broader implications.

Is the work clearly and accurately presented and does it cite the current literature?

Yes

If applicable, is the statistical analysis and its interpretation appropriate?

Not applicable

Are all the source data underlying the results available to ensure full reproducibility?

Yes

Is the study design appropriate and is the work technically sound?

Yes

Are the conclusions drawn adequately supported by the results?

Yes

Are sufficient details of methods and analysis provided to allow replication by others?

Partly

Reviewer Expertise:

Social Psychology, Positive Psychology, Education

I confirm that I have read this submission and believe that I have an appropriate level of expertise to confirm that it is of an acceptable scientific standard, however I have significant reservations, as outlined above.

## References

[1] : A mixed method study on Chinese primary school EFL teachers' preparation, affecting factors and support needed to implement intercultural foreign language teaching. *PLoS One* .2023;18(4) : 10.1371/journal.pone.0284146 e0284146 10.1371/journal.pone.0284146 37027439 PMC10081789

[2] : Emergency remote learning during COVID-19 crisis: Students' engagement. *Educ Inf Technol (Dordr)* .2021;26(6) : 10.1007/s10639-021-10566-4 7033-7055 10.1007/s10639-021-10566-4 33935578 PMC8077864
